# Supplementary figures and images for: Comparative genomic analysis of emerging non-typeable Haemophilus influenzae (NTHi) causing emerging septic arthritis in Atlanta
Source: PeerJ. 2025 Mar 21;13:e19081. doi: 10.7717/peerj.19081 (PMC11932112; doi:10.7717/peerj.19081)

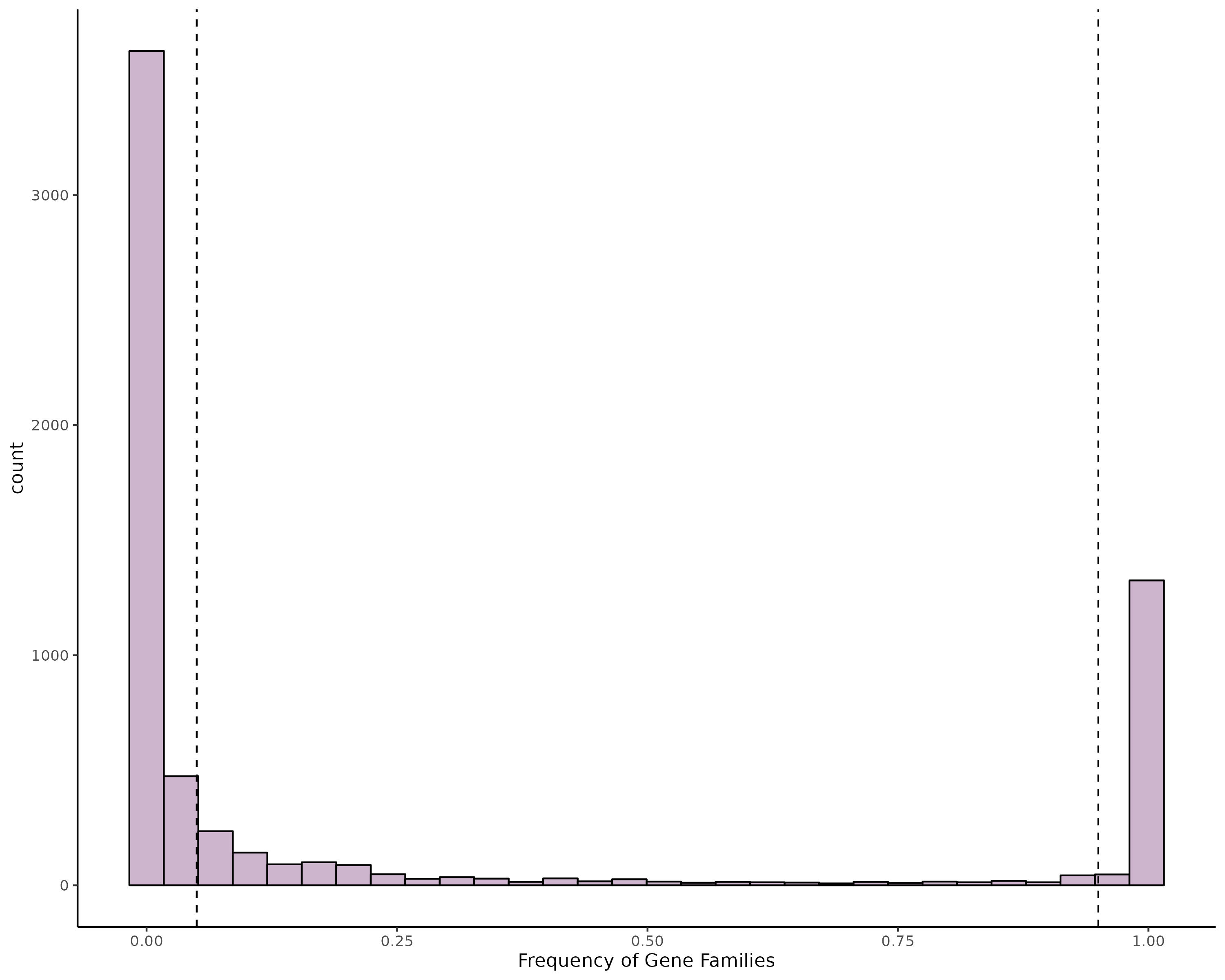

Supplement: Supplemental Information 1 — Histogram representing the percentage of the 6,560 gene families in each of the 583 samples, of which 1368 were core (>= 95% genomes), 1107 intermediate accessory (95% < x <= 5 %) and 4085 rare accessory (x > 5%). [file peerj-13-19081-s001.png]

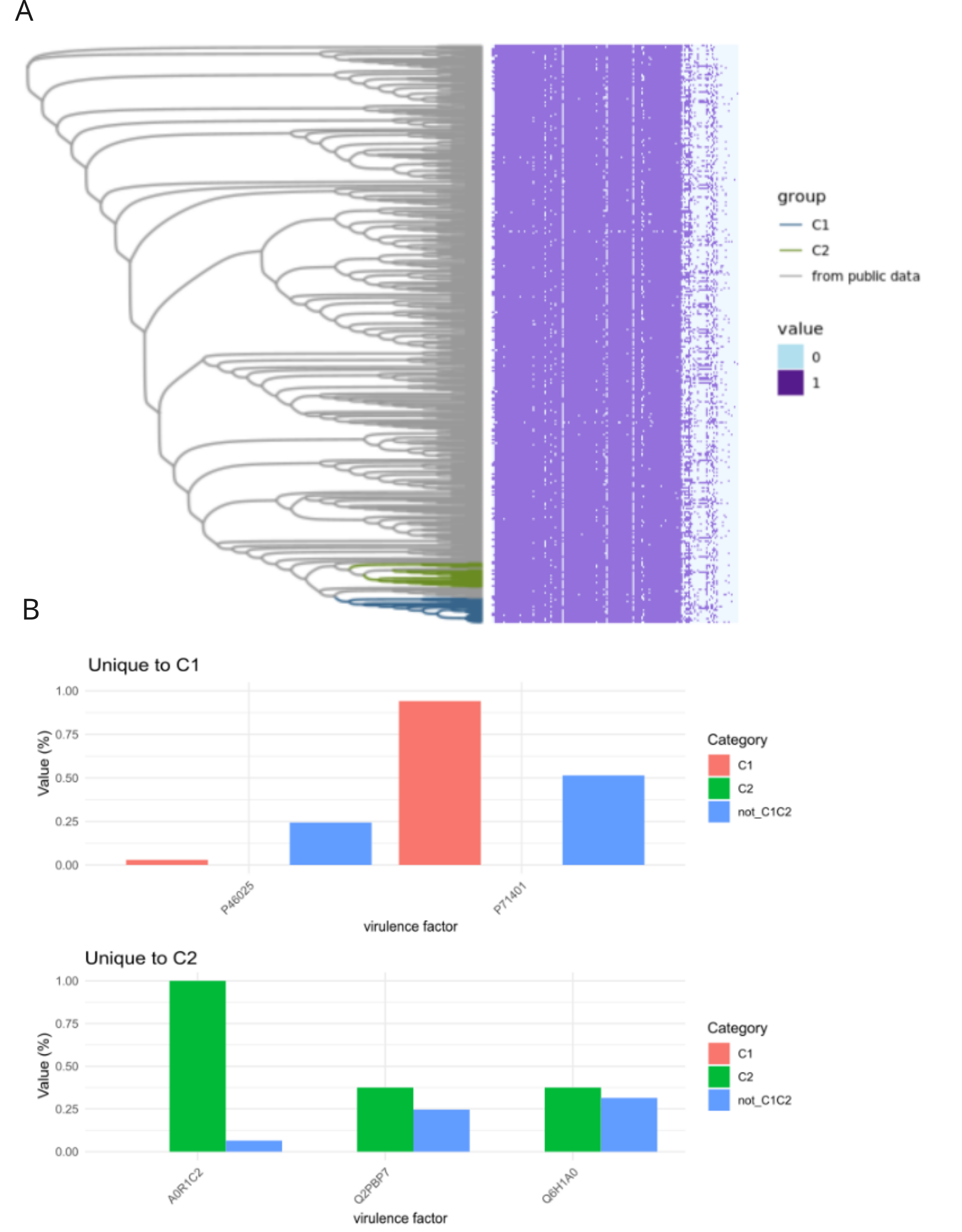

Supplement: Supplemental Information 2 — Results from virulence factor analyses. (A) Phylogenetic tree representing samples from the public data, C1, and C2 with heatmap of presence or absence of virulence factors from blastp against custom virulence gene database. (B) Bar charts of virulence factors common to C1 or C2. The y axis represents the percentage of samples that have the displayed virulence factor. [file peerj-13-19081-s002.png]

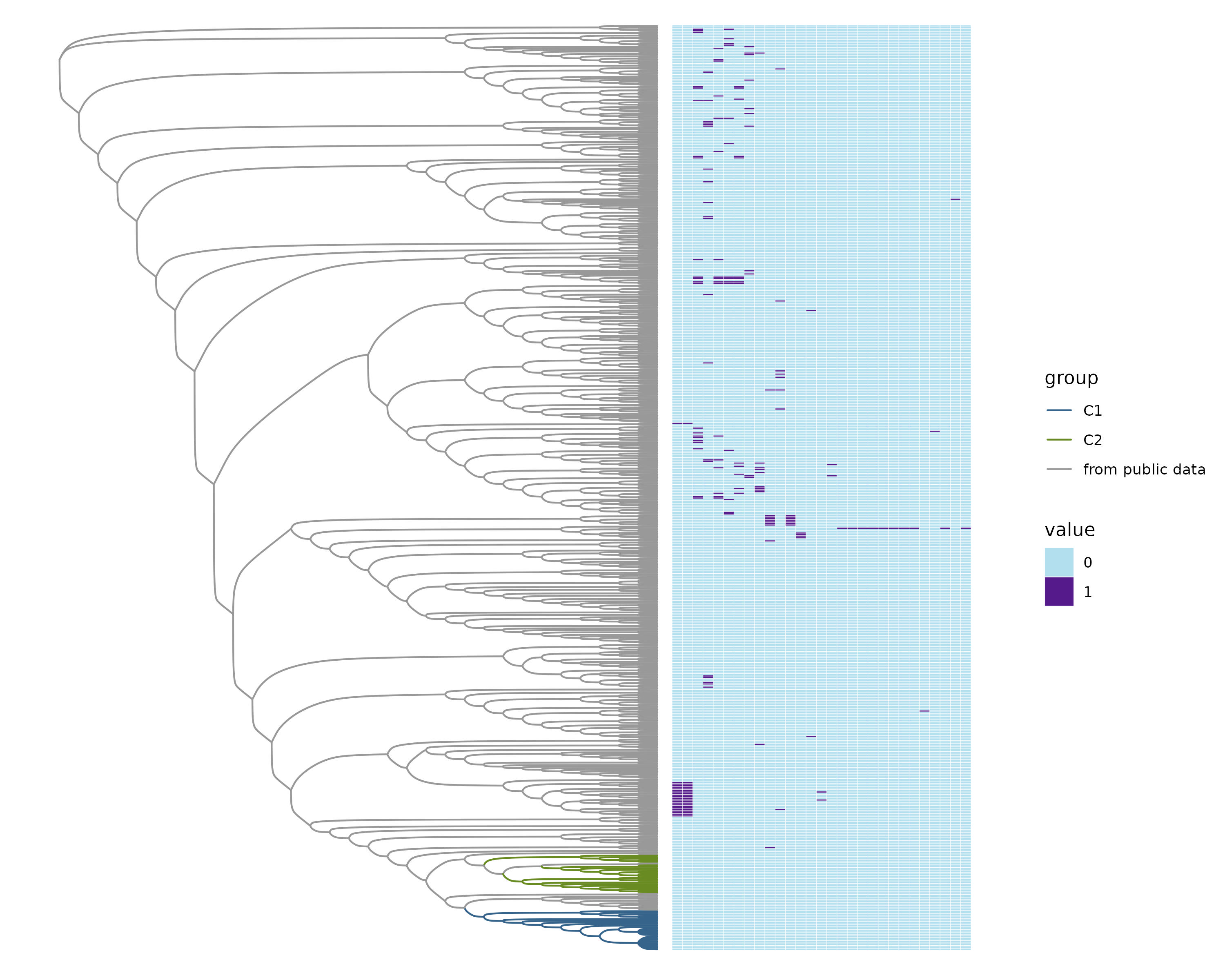

Supplement: Supplemental Information 3 — Phylogenetic tree representing samples from the public data, C1, and C2 next to a heatmap with presence or absence of antimicrobial resistance genes. [file peerj-13-19081-s003.png]

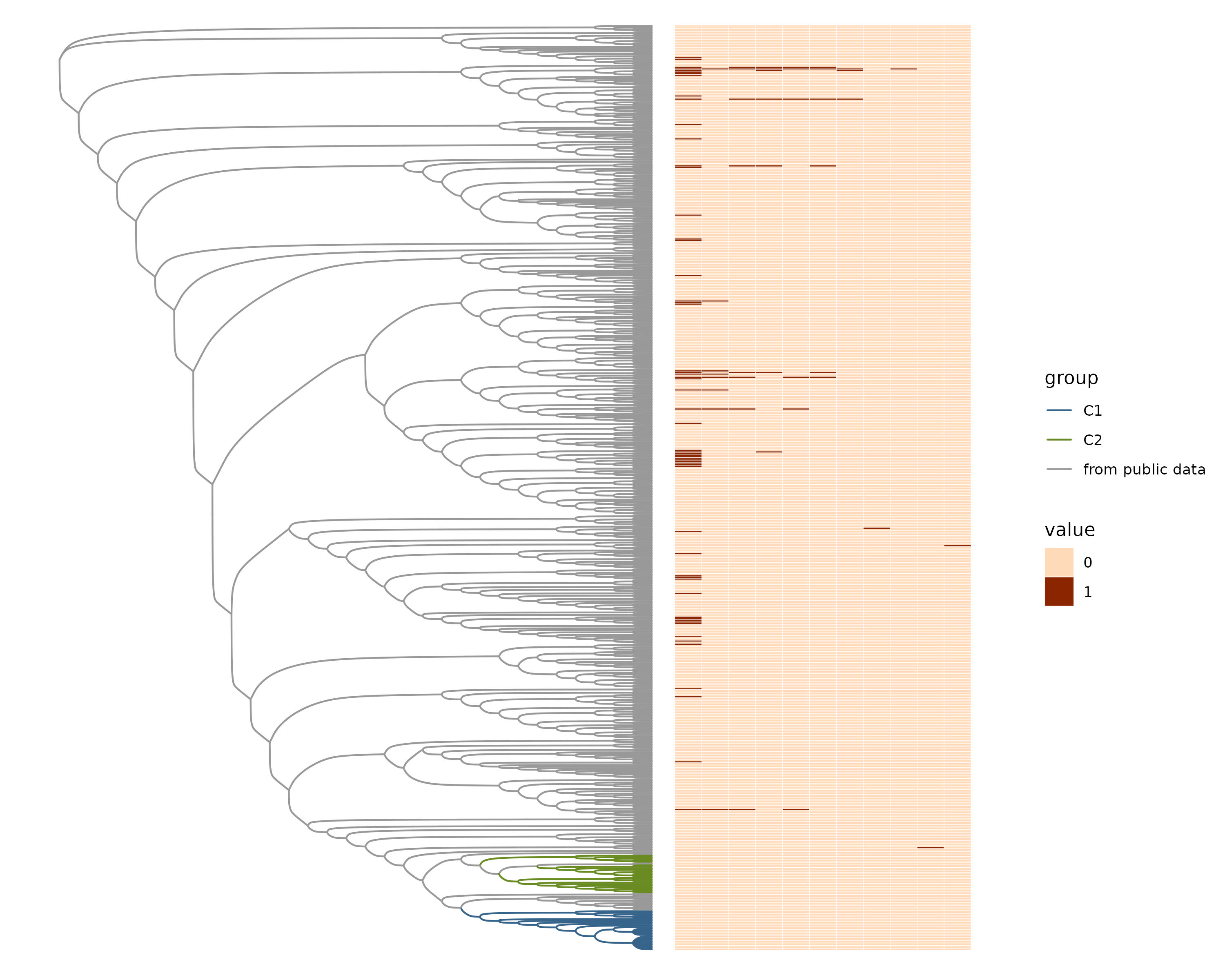

Supplement: Supplemental Information 4 — Phylogenetic tree representing samples from the public data, C1, and C2 next to a heatmap with presence or absence of virulence factors from the custom database in the rare pangenome. [file peerj-13-19081-s004.png]

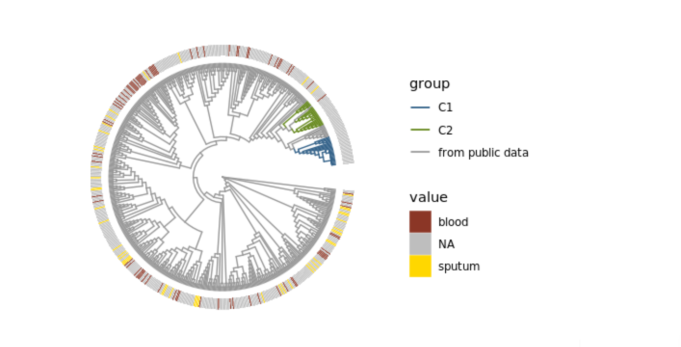

Supplement: Supplemental Information 5 — Phylogenetic tree representing samples from public data, C1, and C2 plotted in grey, blue, and green, respectively. Value legend indicates whether sample was collected from blood, sputum, or whether metadata was not available. [file peerj-13-19081-s005.png]
